# Systematic Development of Self-Nanoemulsifying Liquisolid Tablets to Improve the Dissolution and Oral Bioavailability of an Oily Drug, Vitamin K1

**DOI:** 10.3390/pharmaceutics10030096

**Published:** 2018-07-18

**Authors:** Yongtao Tong, Yuli Wang, Meiyan Yang, Jiahui Yang, Lu Chen, Xiaoyang Chu, Chunhong Gao, Qian Jin, Wei Gong, Chunsheng Gao

**Affiliations:** 1 State key Laboratory of Toxicology and Medical Countermeasures, Beijing Institute of Pharmacology and Toxicology, Beijing 100850, China; tongxiyetao@163.com (Y.T.); wangyuli764@126.com (Y.W.); ymyzi@163.com (M.Y.); jiahui_yang05@yeah.net (J.Y.); c670406@163.com (L.C.); cxy15010248773@163.com (X.C.); 13521775490@163.com (C.G.); 2 Pharmaceutical College, Henan University, Kaifeng 475001, China; jinqian160@163.com

**Keywords:** vitamin K1, self-emulsifying, liquisolid technology, oral drug delivery, dissolution, bioavailability

## Abstract

The purpose of this study is to improve the dissolution and oral bioavailability of an oily drug, vitamin K1 (VK1) by combination of self-nanoemulsifying and liquisolid technologies. The optimal liquid self-nanoemulsifying drug delivery systems (SNEDDS) formulation including VK1 (oil), mixture of soybean lecithin and glycocholic acid (surfactant) and Transcutol HP (cosurfactant) was obtained according to ternary phase diagrams and a central composite design. Based on compatibility, adsorption capacity and dissolution profile, liquid SNEDDS was then solidified on Fujicalin^®^ to form solid SNEDDS by liquisolid technology and compressed directly with excipients into self-nanoemulsifying liquisolid (SNE-L) tablets. Uniform nano-emulsion suspension was formed rapidly when the SNE-L tablets disintegrated in dissolution media and higher drug dissolution was observed compared with the conventional tablets. The results of pharmacokinetic study in beagle dogs showed that the mean *C*_max_ and the area under the curve of SNE-L tablets were remarkably higher than those of conventional tablets, which were consistent with the results of the in vitro dissolution. The relative bioavailability of SNE-L tablets and conventional tablets was approximately 200%. In conclusion, this combination method showed promise to improve the dissolution and oral bioavailability of oily drug vitamin K1.

## 1. Introduction

Vitamin K1 (VK1) is a type of oily drug that is given to treat or prevent excessive bleeding in people with insufficient VK1 or who have taken excessive coumarin anticoagulant medication [1]. VK1 is mainly administered intramuscularly or intravenously; these routes have rapid effects, but they occasionally result in allergic reactions, anaphylactic shock, or hemolysis [2]. To overcome these adverse reactions, a new formulation of VK1 which was called Konakion^®^ MM was launched in 1991; Konakion^®^ MM is based on mixed micelles of lecithin and glycocholic acid [3]. Compared with classical products containing synthetic solubilizers such as Cremophor EL, tween, and Pluronics, Konakion^®^ MM is a safe and effective treatment with excellent biocompatibility for both oral and intravenous administration. However, for better treatment compliance and convenience of using, a solid formulation would be useful.

The low aqueous solubility of poorly water-soluble drugs often leads to poor oral bioavailability because of their slow and limited release of drug in gastrointestinal fluid [4]. Different strategies can be used to address this problem, including the use of solid dispersions, micronization, complexation with cyclodextrins, and nanoparticles. Recently, lipid-based formulations, especially self-emulsifying drug delivery systems (SEDDS), have been widely accepted as a promising approach for delivering poorly water-soluble drugs. A SEDDS is an isotropic pre-concentrate consisting of oil, surfactant, cosurfactant, and drug. The SEDDS spontaneously forms thermodynamically stable oil-in-water emulsions by gentle agitation in the presence of an aqueous phase. When the droplet size of the emulsion ranges between 20 and 200 nm, the SEDDS is a self-nanoemulsifying drug delivery system (SNEDDS) [5]. Poorly water-soluble drugs are dissolved in small oil droplets such that the dissolution process is no longer the rate-limiting step of in vivo absorption [6]. Moreover, a SNEDDS enhances the oral bioavailability of hydrophobic drugs by avoiding the hepatic first-pass effect, reducing metabolism by the cytochrome-P450 family of enzymes, inhibiting P-glycoprotein (P-gp) efflux, and promoting drug transport via lymph circulation [7]. As a liquid formulation, however, inherent defects, such as, migration of the components, potential drug leakage, low stability during manufacturing, have limited its practical industrial application [8]. To overcome these difficulties, solid SNEDDS (S-SNEDDS) formulations have been investigated as an alternative approach.

S-SNEDDS formulations, which can be prepared by spray drying, spray congealing, liquisolid technique, melt granulation, freeze drying technique and extrusion/spheronization, transform lipid formulations into various solid counterparts with the added advantage of convenient process control [9]. Among these methods, liquisolid technique is a convenient and economical way to transform liquid to solid powders without influencing the self-emulsifying properties [10]. Liquisolid systems refer to formulations formed by conversion of liquid drugs, drug suspensions or drug solution in non-volatile solvents into dry, nonadherent, free-flowing, compressible and compactible powder mixtures by blending the suspension or solution with selected carriers and coating materials [11]. Liquisolid systems have been reported to enhance drug release and improve bioavailability owing to increased surface area of the drug, increased aqueous solubility, and improved wettability of drug particles [12,13]. There is little reported literature about the application of the solid self-emulsifying technology in the tablet preparation of cyclosporine A [9], nimodipine [14], CoQ10 [15], and valsartan [16].

In the present study, to enhance solubility and bioavailability of VK1, the self-nanoemulsifying liquisolid (SNE-L) tablets were designed and prepared. The formulation of a liquid SNEDDS (L-SNEDDS) was optimized using a central composite design (CCD) and response surface methodology. The optimal liquid formulation was adsorbed onto suitable carriers and then converted into liquisolid powder. Finally, SNE-L tablets were prepared by direct compression. Uniform nano-emulsion suspension rapidly formed while the SNE-L tablets disintegrated in dissolution media. The dissolution rate in vitro and relative bioavailability in vivo were significantly enhanced by combined application of SNEDDS and liquisolid technology, which can be a useful approach for providing patient-approved dosage form of oily drug VK1.

## 2. Materials and Methods

### 2.1. Materials and Animals

VK1 was purchased from Anhui Wanhe Pharmaceutical Co., Ltd. (Anhui, China). Soy lecithin was purchased from Lipoid GmbH (Ludwigshafen, Germany). Glycocholic acid was purchased from Ark Pharm (Libertyville, IL, USA). Transcutol HP was a kind gift from Gattefosse SAS (Saint Priest Cedex, France). Polyethylene glycol 400 (PEG 400) and propylene glycol were purchased from Sinopharm Chemical Reagent Co., Ltd. (Shanghai, China). Microcrystalline cellulose (Vivapur^®^ PH-102) was purchased from JRS Pharma (New York, NY, USA). Lactose (Cellatose^®^ 80) was purchased from Molkerei Meggle Wasserburg GmbH & Co., KG (Wasserburg, Germany). Anhydrous dibasic calcium phosphate (Fujicalin^®^) and magnesium aluminometasilicate (Neusilin^®^ US2) were generous gifts from Fuji Chemical Industries Co., Ltd. (Tokyo, Japan). Colloidal silicon dioxide (CAB-O-SIL^®^ M-5P) was purchased from Cabot Rheinfelden GmbH (Rheinfelden, Germany). Granulated silicon dioxide (Aeroperl^®^ 300) was obtained as a gift sample from Evonik (Shanghai, China). Sodium carboxymethyl starch, crosslinked sodium carboxymethyl cellulose, crosslinked polyvinylpyrrolidone, and magnesium stearate were purchased from Beijing Fengli Jingqiu Commerce and Trade Co., Ltd. (Beijing, China). High-performance liquid chromatography (HPLC)-grade methanol and acetonitrile were purchased from Sigma Aldrich Trading Co., Ltd. (Shanghai, China). All other reagents were analytical grade.

Beagle dogs were purchased from the Laboratory Animals Center of Beijing Institute of Pharmacology and Toxicology (Beijing, China). All animals were handled according to the code of ethics in research, training, and testing of drugs established by the Animal Care and Use Ethics Committee of the Beijing Institute of Pharmacology and Toxicology (Certificate number: 2017041).

### 2.2. Determination of Drug Concentration

The drug concentration was determined by HPLC analysis with a Waters Alliance HPLC system (UV detector 2487, Separations module 2695, Waters, Milford, MA, USA). Chromatographic separation was accomplished using a reversed phase C_18_ analytical column (Capcell core C_18_, 2.1 × 100 mm, 2.7 μm, Shiseido Co., Ltd., Tokyo, Japan). The concentration of VK1 was measured by absorbance at 254 nm with a column heater set to 25 °C. The mobile phase consisted of a mixture of methanol, acetonitrile, and water in the ratio 88:10:2. The flow rate of the mobile phase was 0.3 mL/min, and a 10-μL sample was injected.

### 2.3. Preparation and Characterization of L-SNEDDS

#### 2.3.1. Formulation Screening of L-SNEDDS

Ternary phase diagrams were used for the formulation screening of L-SNEDDS which obtained the optimal oil phase, surfactant, and cosurfactant for self-emulsification. In this study, water-insoluble VK1 was used as the oil phase. Soybean lecithin and glycocholic acid were used together as surfactants, and Transcutol HP, polyethylene glycol 400 (PEG 400), and propylene glycol were selected as candidates for cosurfactants. The oil phase, surfactant, and cosurfactant were set as three vertices of the ternary phase diagram. All materials were weighed and mixed in accordance with specific points representing different component proportions in the ternary phase diagram. Each formulation was magnetically stirred in a water bath at 60 °C until a yellow transparent liquid self-emulsifying drug solution formed. Then, 1 mL of the L-SNEDDS was added to 100 mL distilled water (37 °C) under constant magnetic stirring to evaluate the self-emulsifying peformance. The self-emulsifying results and the appearance of the generated emulsion (clear, light blue or opalescent) were recorded and plotted in the phase diagrams. Then, the effective self-emulsifying region was plotted to select the type of cosurfactant and the optimal ratio of the two surfactants.

#### 2.3.2. Formulation Optimization of L-SNEDDS

The central composite design and response surface methodology were adopted to optimize the formulation of the L-SNEDDS. Based on the results of ternary phase diagram, the weight percent of oil (X_1_, ranging from 15% to 30%) and the weight ratio of surfactant to cosurfactant (K_m_; X_2_, ranging from 0.6 to 3) were set as two factors. Thus, a central composite design with two-factor and five-level (−a, −1, 0, 1, a) (Table 1) was selected. The droplet size (DS), zeta potential, and drug dissolution efficiency after 20 min (DE_20min_) were selected as three responses.

#### 2.3.3. Characterization of L-SNEDDS

• Dispersibility Test

The dispersibility test was performed to observe the phase clarity of the emulsions and self-emulsification time. One milliliter of the optimized L-SNEDDS was added to 100 mL distilled water (37 °C) with gentle agitation to assess emulsion formation and the appearance of the formed nanoemulsion.

• Robustness to Dilution

Different dilution ratios and diluents might significantly affect the physical stability of the spontaneously emulsifying systems. Accordingly, nanoemulsions diluted to different proportions (10-, 100-, and 1000-fold) with different diluents (purified water, 0.1 N HCl, phosphate buffer pH 6.8, and 0.5% SDS aqueous solution), and then stored for 24 h at room temperature for physical stability study.

• Droplet Size, Zeta Potential and Medium Viscosity

The optimized L-SNEDDS was diluted with distilled water (1:100) under constant magnetic stirring. The droplet size (DS), polydispersibility index (PDI), and zeta potential of the droplets were then determined by dynamic light scattering using a Malvern Zetasizer (Nano ZS-90, Malvern instruments Ltd., Malvern, UK). The medium viscosity of the solutions were achieved with a viscometer (Fungilab, VISCOLEAD, Co., Ltd., Barcelona, Spain). All values recorded were the average of three measurements.

• In Vitro Dissolution Studies

The in vitro dissolution profile of L-SNEDDS was assessed using the USP paddle method with a USP dissolution apparatus (SR8PLUS dissolution tester, Hanson Research Corporation, Chatsworth, CA, USA) in 500 mL water at 100 rpm and 37 ± 0.5 °C. Hard capsules were each filled with an L-SNEDDS containing 10 mg VK1 and placed in dissolution media. Aliquot samples of 5 mL were withdrawn at certain time intervals (5, 10, 20, 30 min) and filtered using a 0.45 μm glass microfiber filter. The filtered samples were diluted with ethanol and the drug concentration was measured spectrophotometrically using a Shimadzu-1750 UV-visible spectrophotometer (Shimadzu, Japan) and the absorbance had a λ_max_ of 273 nm.

• Transmission Electron Microscopy (TEM)

TEM was used to evaluate the morphology of the L-SNEDDS emulsion droplets. The optimized L-SNEDDS was diluted using distilled water (1:50) with constant magnetic stirring. A drop of nanoemulsions was placed on a copper grid coated with carbon film and then stained with 3% (*w*/*v*) phosphotungstic acid solution for 2 min, and then observed under a transmission electron microscope (Hitachi H-7650, Tokyo, Japan) while the grid was air-dried at room temperature.

### 2.4. Preparation and Characterization of S-SNEDDS

#### 2.4.1. Screening of the Adsorbing Agents

The liquisolid technology was used to solidify the L-SNEDDS to S-SNEDDS. Different porous adsorbents including Vivapur^®^ PH102, Cellatose^®^ 80, Fujicalin^®^, Neusilin^®^ US2, Cab-O-Sil^®^ M5P, and Aeroperl^®^ 300 were used to absorb the L-SNEDDS.

Firstly, Scanning electron microscopy (SEM) was utilized in order to assess the morphological characteristics of the adsorbents using a Hitachi (S4800, Tokyo, Japan) scanning electron microscope. The samples were coated with a thin gold layer and examined in the microscope using an accelerating voltage of 15 kV.

Secondly, a compatibility test was performed to investigate the influence of solid adsorbents on the stability of the drug. Accurately weighed samples of the L-SNEDDS (100 mg) and each of selected adsorbents (200 mg) were mixed by co-grinding them manually in an agate mortar to obtain non-sticky solid powder. Then, the liquid-solid mixture was placed in an oven at 60 °C/92% RH protected from light for 10 days. The total impurity contents of the samples were analyzed by HPLC after 1, 5, and 10 days to evaluate the compatibility of the drug and adsorbents.

Finially, the liquid adsorption capacity of each type of adsorbent was evaluated. However, preparation of a liquisolid with desired flow rate and compressibility was possible when the amount of the carrier was appropriate. The maximum amount of liquid was termed as liquid load factor (*L_f_*). The value of *L_f_* was calculated as follows:

(1) $$L_{f}=\Phi_{\mathrm{ca}}+\Phi_{\mathrm{co}}\times \frac{1}{R}$$

where Φ_ca_ and Φ_co_ were carrier flowable liquid-retention potential (Φ-value) and coating Φ-value, respectively. R represented the ratio between the carrier materials and the coating materials. In order to get the optimal flowable liquid-retention potential for adsorbents, an experiment was designed to measure angle of slide. Use 10 g adsorbent to adsorb L-SNEDDS, and put the admixture on a metal plate which was polished. Thentilte the plategradually, the angle of the plate was the angle of slide (θ) when the admixture slid. The Φ-value was calculated based on the following equation. The Φ-value at 33° was reported to represent the optimal Φ-value of the adsorbent in L-SNEDDS [17].

(2) $$\Phi -\mathrm{value}=\frac{\mathrm{weight}\;\mathrm{of}\;L-\mathrm{SNEDDS}}{\mathrm{weight}\;\mathrm{of}\;\mathrm{adsorbents}}$$

#### 2.4.2. Preparation of S-SNEDDS

Then the liquisolid powder was prepared using L-SNEDDS as liquid medication and selected adsorbent as both the carrier and coating material. Determine the amount of L-SNEDDS(W) required based on the drug concentration in L-SNEDDS to ensure that each SNE-L tablet contains 10 mg VK1. According to the Equations (3) and (4) [18], the amounts of the carrier (Q) and the coating (q) can be obtained. For all liquid solid systems, the carrier: coating ratio (R) was chosen to be 20.

(3) $$L_{f}=\frac{W}{Q}$$

(4) $$R=\frac{Q}{q}$$

Then was incorporated the calculated quantitiesad sorbent with the appropriate amount of L-SNEDDS using the way of Spireas et al. [19]. The prepared liquisolid powder was yellow uniform granules with good flowability.

#### 2.4.3. Characterization of Angle of Repose

The angle of repose was measured by the fixed height funnel method. Powder with a lower angle of repose showed better flow properties due to decreased adhesion. The height (*h*) and radius (*r*) of the powder cone were recorded after the mixture passed through the funnel, and the angle of repose (𝜃) was calculated using the following equation:

(5) $$\;\theta =tan^{-1}\frac{h}{r}\;$$

• Carr’s Index

The flowability of all liquisolid powders was assessed based on Carr’s compressibility index (*CI*), which is derived from the tapped density (*P_t_*) and poured bulk density (*P_b_*) of the liquisolid powders. The prepared liquisolid powders were weighed and poured into a graduated cylinder and the Pb and Pt can be obtained from poured bulk volume (*V*b) and the tapped volume (*V*t). Then the *CI %* was calculated as follows [20]:

(6) $$\;CI\;\%=\frac{P_{t}-P_{b}}{P_{t}}\times 100\;$$

• Hausner’s Ratio

The Hausner ratio was calculated as the quotient of tapped density (*P_t_*) and bulk density (*P_b_*).

(7) $$HR=\frac{P_{t}}{P_{b}}$$

### 2.5. Preparation and Characterization of Self-Nanoemulsifying Liquisolid (SNE-L) Tablets

#### 2.5.1. Preparation of SNE-L Tablets

The liquisolid powder was blended with fillers, disintegrants and lubricants and then compressed into a tablet in a single-punch tablet press (Shanghai Tianhe Pharmaceutical Device Co., Ltd., Shanghai, China) using a 10-mm diameter punch. Conventional VK1 tablets were prepared by the same procedure of SNE-L tablets except VK1 replaced the L-SNEDDS. Each tablet weighed 400 mg and contained 10 mg VK1.

#### 2.5.2. Characterization of SNE-L Tablets

• In vitro Dissolution Studies

The in vitro dissolution profile of SNE-L tablets was assessed as described in the section of Liquefaction Time. The distilled water, pH 1.0, hydrochloric acid solution, pH 6.8 phosphate buffer solutions and 0.5% sodium dodecyl sulfate (SDS) solution were used as dissolution media separately.

• Liquefaction Time

One tablet of the optimum formulation was wrapped in a transparent polyethylene film and tied to the bulb of a thermometer with thread. The thermometer with attached tablet was placed in a dissolution vessel containing 500 mL dissolution media and maintained at 37 ± 0.5 °C. The tablet was observed carefully, and the melt time was recorded. An average of 10 measurements were performed for each aqueous medium.

• Physical Characterization Tests

SNE-L tablets were evaluated for hardness and friability. Hardness was measured using a manual hardness tester (Eyweka). The friability (Fr) was determined (*n* = 3) using a friabilator (TA, Erweka, Heusenstamm, Germany). Twenty accurately weighed tablets (*M*1) were placed in the drum, and the sample was subjected to falling shocks for 4 min at a rotational speed of 25 rpm. Then, the weight of the tablets after the test (*M*2) was accurately measured and used to calculate the friability by the following equation:

(8) $$\mathrm{Fr}\left(\%\right)=\left[\frac{M1-M2}{M1}\right]\times 100$$

• Reconstitution Study

A SNE-L tablet (400 mg) was dispersed in a volumetric flask containing 25 mL of distilled water by shaking gently. The resulting suspension was incubated for 30 min at room temperature to completely precipitate excipients, and the suspension was then filtered through a 0.45-μm filter. The DS and PDI of the emulsion were then determined.

### 2.6. In Vivo Pharmacokinetic Study

#### 2.6.1. Drug Administration

A single-dose, randomized, two-period crossover design for pharmacokinetic study with a washout period of a week was carried out. Six healthy beagle dogs (9.9 ± 0.8 kg) were randomly divided into two groups (SNE-L tablets and conventional VK1 tablets). The dogs were fasted but had free access to water overnight. Each dog received either a single 10 mg SNE-L tablet or a single 10 mg conventional tablet. Dogs were giver free access to water and food 6 h after drug administration.

#### 2.6.2. Blood Sampling

Serial blood samples (2 mL) were collected in heparinized tubes using an indwelling cannula before dosing and at 0.25, 0.5, 0.75, 1, 2, 3, 4, 5, 6, 8, 10, 12, and 24 h after dosing. Blood samples were cooled in an ice bath and centrifuged at 3000 rpm for at least 10 min at 4 °C. The harvested plasma was stored at −70 °C until analysis. The isolation process was performed with protection from light.

#### 2.6.3. Analytical Methods

The VK1 concentration in dog plasma was quantified using a 1260 Infinity high-performance liquid chromatographic (HPLC) and G6460A triple-quad mass spectrometer (Agilent Technologies, Santa Clara, CA, USA). Fenofibrate was used as the internal standard. The HPLC-MS/MS analysis conditions were as follows: Agilent Zorbax Eclipse XDB C_8_ analytical column (2.1 × 100 mm, 3.5 μm, Agilent Technologies, Santa Clara, CA, USA); mobile phase, methanol containing 5 mM ammonium formate; flow rate, 0.3 mL/min; multiple-reaction monitoring (MRM) in negative ionization mode; transitions *m/z*, 451.3 → 187.0 for VK1 and 361.1 → 233.0 for fenofibrate; fragment voltage, 140 V and 135 V for VK1 and fenofibrate, respectively; capillary voltage, 4000 kV; gas temperature, 300 °C; gas flow, 11 L/min; nebulizer pressure, 25 psi.

#### 2.6.4. Pharmacokinetic and Statistical Analysis

The pharmacokinetic analysis of both VK1 tablets was performed using a non-compartmental model. *C*_max_ (the highest observed plasma concentration) and *T*_max_ (the time required to reach *C*_max_) were observed from the individual plasma concentration-time curves. The area under the curve (AUC) was calculated using the linear trapezoidal rule. The half-life (*t*_1/2_) was calculated from the slope of the logarithm of concentration vs. time profile. The relative availability (*F*_rel_) was calculated as (*AUC_T_*/*AUC_R_*) × 100%.

A t-test was employed to compare the pharmacokinetic parameters between two formulatios. ANOVA was performed on un-transformed and log-transformed data for *C_max_* and AUC. All testings were performed by SPSS 16.0 (SPSS Inc., Somers, New York, NY, USA).

## 3. Results and Discussion

### 3.1. Factors Affecting Nanoemulsions Region in Phase Diagrams

**Figure 1:**
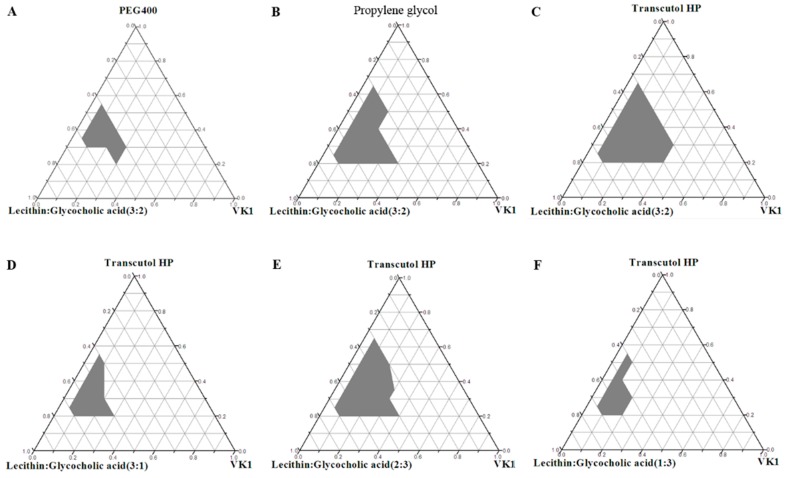
Ternary phase diagrams for oil (vitamin K1), cosurfactant (PEG400, propylene glycol, Transcutol HP), and 3:1, 3:2, 2:3, 1:3 ratios of mixed surfactants (soybean lecithin and glycocholic acid).

Since VK1 was miscible with the commonly used surfactant and cosurfactant, VK1 was used directly as the oil phase instead of using other oils. The ternary phase diagrams of oil (VK1), surfactants (mixture of soybean lecithin and glycocholic acid), and cosurfactant (Transcutol HP, PEG 400, or propylene glycol) were constructed (Figure 1) to optimize the L-SNEDDS formulation.

First, the phase diagrams of the system containing Transcutol HP, PEG 400, or propylene glycol as the cosurfactant with mixture of soybean lecithin and glycocholic acid (3:2) as surfactants were plotted in Figure 1A–C. The largest nanoemulsion region was obtained when Transcutol HP was used as the cosurfactant (Figure 1C). This result may occur because Transcutol HP decreases the bending stress of the interface and forms a more flexible interfacial film with surfactants [21]. In addition, Transcutol HP has a relatively low hydrophilic lypophilic balance (HLB) and can load a large amount of oil with stronger lipophilicity [22]. Therefore, according to the self-emulsification domains based on the ternary phase diagrams, Transcutol HP was used as the cosurfactant in the SNEDDS.

Natural surfactants (including lecithin, cholalic acid, and Peceol) are preferred due to their more favorable safety profiles. However, the self-emulsification efficiency of these natural surfactants is not enough [23]. To achieve appropriate HLB value and improve self-emulsifying ability, combination of different surfactants is commonly used [24]. In the present study, soybean lecithin and glycocholic acid were used together as mixed surfactants and the results are shown in Figure 1. From the results, the maximum nanoemulsion region was obtained when the mixing ratio of soybean lecithin and glycocholic acid was 3:2. Therefore, based on these results, VK1 (oil phase), Transcutol HP (cosurfactant) and soybean lecithin/glycocholic acid (3:2, *w*/*w*, surfactants) were selected for L-SNEDDS.

### 3.2. Optimization of the L-SNEDDS Formulations

For the further optimization of the L-SNEDDS compositions, the central composite design and response surface methodology were used and the weight percent of oil and the weight ratio of surfactant to cosurfactant were selected as two factors (Table 2). The droplet size, zeta potential, and DE_20min_ were selected as three responses. Different regression model fittings were generated by determining the droplet size zeta potential and DE_20min_. The ANOVA results indicated that the relationship between responses and factors fit a quadratic equation well. The values of *R*^2^ were all above 0.9861, and simultaneously the fitting significance values of *p* were all less than 0.01 (Table 3).

.

**Figure 2:**
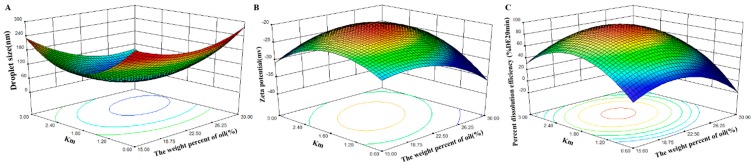
Response surface plot (3D) of droplet size (**A**); zeta potential (**B**); and percent drug dissolution efficiency after 20 min (**C**).

**Figure 3:**
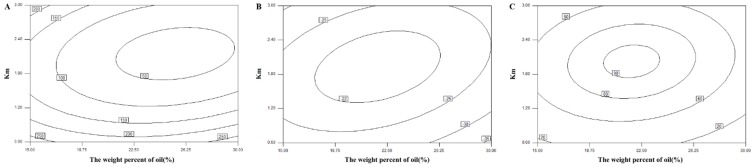
Contour maps of droplet size (**A**); zeta potential (**B**); and percent drug dissolution efficiency after 20 min (**C**).

According to the regression equation, the respective 3D-response surfaces and contour maps were depicted per response variable (Figure 2 and Figure 3). The droplet size of formed emulsions plays an important role because it determines the release characteristics and the bioavailability of the drug [25]. In the 13 experiments described above, the droplet size was mostly on the nanometer scale ranging from 50 to 250 nm. As shown in Figure 2A and Figure 3A, when the weight ratio of surfactant to cosurfactant (K_m_) was maintained at a low level, no obvious difference in the droplet size was observed with changes of oil phase. Therefore, K_m_ was considered a vital factor in effecting self-emulsifying ability. When the weight percent of oil phase was lower, the droplet size of emulsions showed an initial decrease as K_m_ increased from 0.6 to 2.0, which was followed by a gradual increase. This result occurs because the emulsifying ability was originally enhanced by the increasing amount of surfactant, which led to a smaller mean droplet size. Then, the trend reversed due to the interfacial disruption elicited by enhanced water penetration into the oil droplets mediated by the increased surfactant concentration; this led to the ejection of oil droplets into the aqueous phase [26]. Moreover, the high viscosity of the L-SNEDDS formulation, which was attributed to the increasing surfactant concentration, also inhibited the self-emulsifying process and increased the droplet size. However, when the weight percent of the oil phase with smaller viscosity was higher, it could limit the increase of the total viscosity of the L-SNEDDS formulation, and thus the droplet size of the emulsion continuously decreased with the increase of K_m_ value [27].

Zeta potential, which indicates the interaction force between charged particles, could influence the physical stability of emulsions by preventing the coalescence of particles. It was reported that a modest potential value has a favorable stabilizing effect on the particle dispersion system [28]. As shown in Figure 2B and Figure 3B, the evaluated formulations showed negative potential that can be attributed to the ionic surfactants. When the K_m_ was too small or too large, the self-emulsifying ability of the liquid SNEDDS was hampered, which increased the level of interfacial free glycocholic acid. Thus, the negative potential value of the nanoemulsion decreased [29].

The drug dissolution efficiency of the L-SNEDDS was measured by quantifying the loaded drug in fine emulsion droplets. Thus, the dissolution test of the SNEDDS formulation could also be regarded as an indirect reflection of self-emulsifying capacity and efficiency. Figure 2C revealed that the variation trend of DE_20min_ was similar to that of the zeta potential. To ensure a high dissolution rate of VK1, a DE_20min_ value higher than 90% was considered a key attribute for optimization.

**Figure 4:**
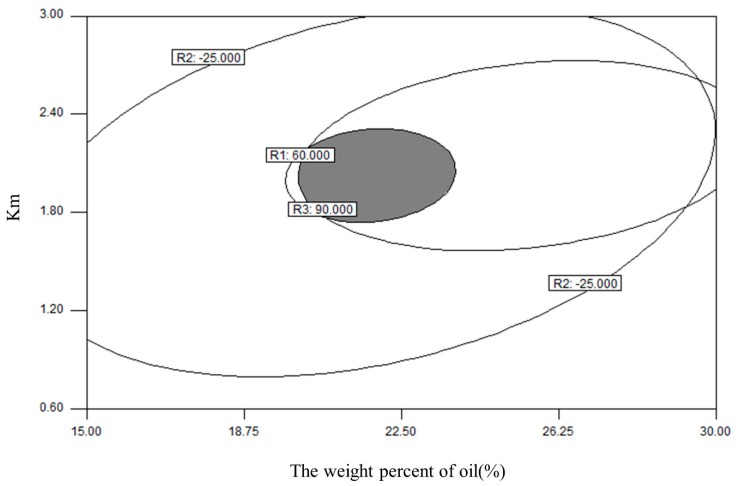
The overlapping contour maps of droplet size (R1) and percent drug dissolution efficiency after 20 min (R3). The gray shadow represents the optimum region for L-SNEDDS formulations (R1 < 60 nm, R3 > 90%).

Considering that the zeta potential of all the formed nanoemulsions were in the reasonable range of −30 to −20 mV and varied slightly, the contour maps of the other two parameters were overlapped to form the optimized region of the final formulation (Figure 4), in which nonoemulsions could be formed with a DS smaller than 60 nm and a DE_20min_ higher than 90%. There was greater theoretical and practical value when the L-SNEDDS had relatively high drug loading. Moreover, the low viscosity of formulations favored the subsequent solidification process. Thus, the L-SNEDDS with X1 = 22.5 and X2 = 1.8 was selected as the optimized formulation for further development of a S-SNEDDS. A random liquid formulation (X1 = 20.0, X2 = 2.0) in the optimized area was picked for formulation validation. Comparing observed values and predicted values, all errors are less than 5%, which indicates that the model has satisfactory predictability (Table 4).

### 3.3. Characterization of L-SNEDDS

The L-SNEDDS was yellow oily liquid. After dispersed in water, yellow or yellowish transparent nanoemulsion formed. The dispersibility of L-SNEDDS and the droplet size, zeta potential and TEM image of nanoemulsion were investigated.

Dispersibility is a main indicator to assess the self-emulsification efficiency. In this study, dispersibility tests showed that the optimized L-SNEDDS formulation could form a clear nanoemulsion within 15 s when diluted in different dilution ratios (10-, 100-, and 1000-fold) and with diluents (purified water, 0.1 N HCl, phosphate buffer pH 6.8, and 0.5% SDS aqueous solution). Moreover, the formed nanoemulsions were stable without phase separation in all diluents for 24 h at room temperature.

**Figure 5:**
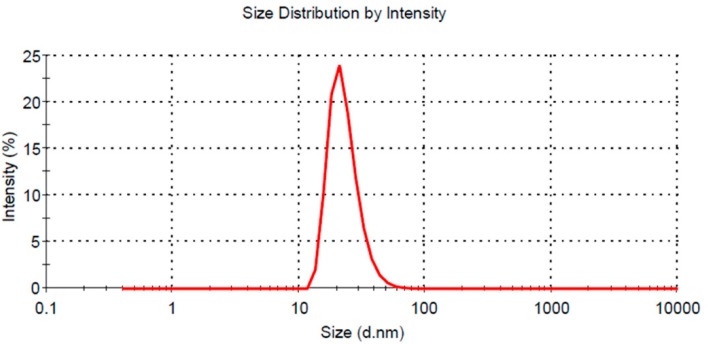
Droplet size distribution of the optimized liquid SNEDDS formulation.

**Figure 6:**
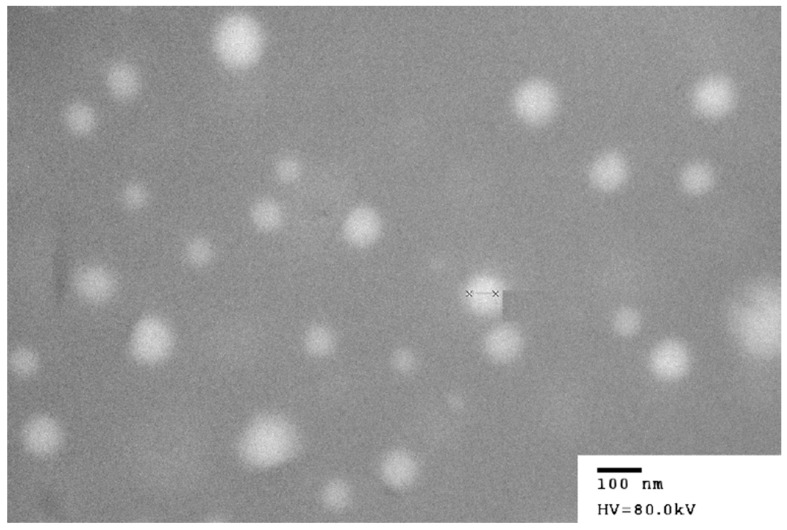
TEM image of the optimized liquid SNEDDS formulation.

The droplet size of the optimal formulation was 47.74 nm with a PDI of 0.248 (Figure 5), which indicated that emulsion droplets were sufficiently small to achieve an excellent release rate and absorption [30]. The TEM image (Figure 6) of the optimized L-SNEDDS confirmed the droplet size results. Dilution ratios had little effect on the particle size of the nanoemulsion. When 1 mL of the L-SNEDDS formulation was diluted 10-, 100-, and 1000-fold with distilled water, the particle size of the nanoemulsion was 50.46 nm, 47.74 nm, and 45.34 nm, respectively, and the PDI values were all less than 0.3. The particle sizes of nanoemulsions diluted with distilled water and 0.1 N HCl were similar, but the particle size in 100 mL phosphate buffer pH 6.8 and 0.5% SDS aqueous solution decreased slightly to 42.65 nm and 39.78 nm, respectively. This result may be because the ions in pH 6.8 PBS have a weak effect on the charged emulsion droplets, and the addition of surfactants in 0.5% SDS aqueous solution also affected the formation of a nanoemulsion.

Zeta potential, which is an important factor for nanoemulsion stability [31], was −20.53 mV, conferring favorable physical stability to the system. At 37 °C, the viscosity of the the L-SNEDDS is 780 cP. But the L-SNEDDS with 100-fold dilution has a viscosity of 48.54 cP.

### 3.4. Effect of Solid Adsorbents on Characterization of Liquisolid Powder

**Figure 7:**
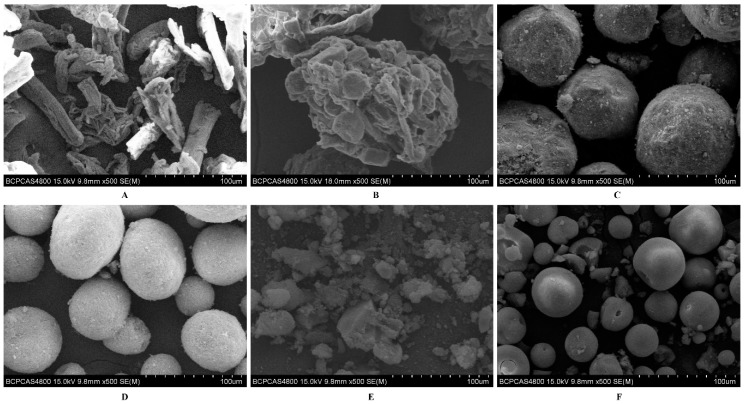
SEM micrographs of various solid adsorbents. (**A**) Vivapur^®^ PH102; (**B**) Cellatose^®^ 80; (**C**) Fujicalin^®^; (**D**) Neusilin^®^ US2; (**E**) Cab-O-Sil^®^ M5P; and (**F**) Aeroperl^®^ 300.

As shown in Figure 7, the investigated solid adsorbents had specific shapes and structures. Microcrystalline cellulose (Vivapur^®^ PH102) and lactose (Cellatose^®^ 80) are commonly used carriers [32]. Fujicalin^®^ is a spherically agglomerated, porous, ultralight anhydrous dibasic calcium phosphate with a large specific surface area (SSA) of 32 ± 1 m^2^/g, which is 32 times higher than that of Vivapur^®^ PH102 (SSA: 1 ± 0 m^2^/g). Neusilin^®^ US2 (SSA: 339 ± 1 m^2^/g), a synthetic amorphous form of magnesium aluminometasilicate, is prepared by spray drying and thus provides large numbers of micropores [33,34]. Based on their high SSAs, these two materials could be used as solid adsorbents while maintaining good flowability and tabletability for direct compression. Colloidal silicon dioxide (Cab-O-Sil^®^ M5P), which was formed by the nanometer-sized primary particles, has become a traditional solid adsorbent due to its high oil adsorption capacity. In contrast, granulated silicon dioxide (Aeroperl^®^ 300) with a mean particle diameter ranging from 30 to 40 μm exhibited a larger pore volume as well as better flowability and compressibility.

**Figure 8:**
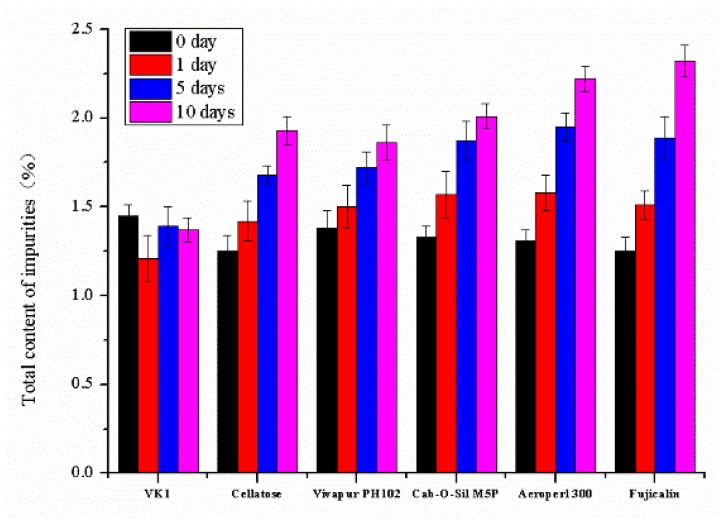
Total content of impurities after incubation at 60 °C/92% RH away from light for 1, 5, or 10 days for drug-excipient chemical compatibility study.

VK1-solid adsorbents’ compatibility at 60 °C with 92% RH was assessed for primary screening (Figure 8). VK1 degraded approximately 69.43% in one day in the presence of Neusilin^®^ US2, but the other five excipients did not remarkably affect the stability of VK1; total impurities of less than 3% were observed in ten days. Thus, Neusilin^®^ US2 should not be used as a solid adsorbent for VK1-SNEDDS.

The liquid adsorption capacity is an important property for carrier and coating materials. Meanwhile, powder flowability is crucial in the industrial production of tablet dosage forms. Therefore, the flowable liquid-retention potential of adsorbents in L-SNEDDS was measured by angle of slide determination and used to calculate the loading factor. The *L_f_* was then used to decide the optimum amount of carrier and coating materials required to ensure dry-looking, free-flowing and compactible powdered systems (Table 5). According to the value of *L_f_*, microcrystalline cellulose (Vivapur^®^ PH102) and lactose (Cellatose^®^ 80) showed the lowest oil adsorption capacity. Fujicalin^®^ had medium adsorption capacity. The absorption quality of Cab-O-Sil^®^ M5P and Aeroperl^®^ 300 showed the best absorption quality due to large specific surface area and porosity. Moreover, all the batches of liquisolid powder showed acceptable flow characteristics with an angle of repose < 45°, Carr’s index less than 35, and Hausner’s ratio less than 1.7.

**Figure 9:**
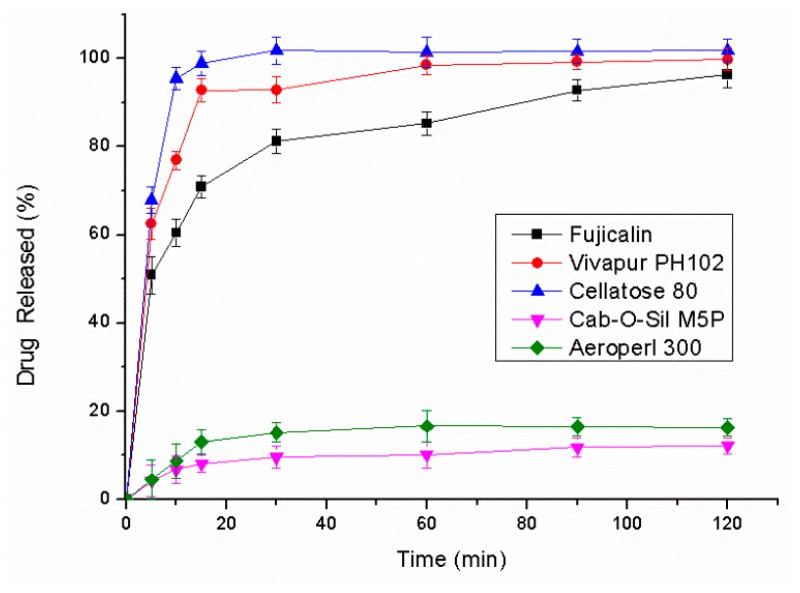
Dissolution profiles of mixtures of the liquid SNEDDS and various solid adsorbents (*n* = 3).

The dissolution profiles of five liquisolid powders were investigated (Figure 9). In addition to Cab-O-Sil^®^ M5P and Aeroperl^®^ 300, drug adsorbed in another three types of carriers was released quickly and almost completely. This result occurred because silicon dioxide had strong adsorbability and hydrophobicity, which may hinder the desorption process of a liquid SNEDDS [27]. Taking into account the low absorption quality of Vivapur^®^ PH102 and Cellatose^®^ 80, Fujicalin^®^ was ultimately selected as the solid adsorbent.

### 3.5. Preparation and Characterization of SNE-L Tablets

#### 3.5.1. Preparation of SNE-L Tablets

The liquisolid powder was blended with fillers, disintegrants and lubricants and then compressed into SNE-L tablet. The type and amount of disintegrating agents, the quantity of lactose, and the effect of colloidal silicon dioxide as a glidant were investigated to ensure rapid and complete dissolution of drug from SNE-L tablets. Based on dissolution profiles, the optimal formulation was found to be composed of liquisolid powder, microcrystalline cellulose, lactose, sodium carboxymethyl starch, colloidal silicon dioxide and magnesium stearate.

Recently, a SNEDDS lyophilized tablets were reported for oral transmucosal delivery of vitamin K. SNEDDS was added to gelatine solution to form nanoemulsion and then freeze-dried [35]. In our research, the L-SNEDDS was solidified by liqusolid method and compressed directly into tablets. The whole process was water-free and need not remove water, which was more economic and stable.

#### 3.5.2. Physical Characterization Tests

The SNE-L tablets and conventional tablets had yellow color with smooth surface. Twenty tablets were randomly selected and weighed individually. The average tablet weight of SNE-L tablets and conventional tablets were 400 mg ± 1.2% and 400 mg ± 1.1% (mean ± coefficient of variation), respectively. The average thickness and hardness of SNE-L tablets and conventional tablets were 44 ± 2 mm/48 ± 3 N and 42 ± 2 mm/67 ± 2 N, respectively. The friability of both tablets were all lower than 0.5%, indicating that the tablets were compact and the surfaces were smooth. The drug contents were 10 ± 0.2 mg (SNE-L tablets) and 10 ± 0.1 mg (conventional tablets).

#### 3.5.3. Liquefaction Time

Considering that the optimum formulation could disintegrate within 5 min in most of aqueous media, the liquefaction times (Table 6) were high compared to disintegration times because agitation was not used in the test. This test was designed to estimate the time it would take the tablets to melt in vivo with no agitation at normal body temperature. In gastrointestinal conditions, however, the gastrointestinal motility will likely decrease the liquefaction time and result in faster emulsification and the penetration of aqueous fluid into the tablet interior [36].

#### 3.5.4. Reconstitution Study

The DS and PDI of the reconstituted nanoemulsion were 51.24 nm and 0.275, respectively. There was no significant difference (*p* > 0.05) compared with the original liquid SEDDS (47.74 nm and 0.248). This result suggested that the solidification process had little effect on the droplet size of the nanoemulsion and the self-nanoemulsifying nature of the SNEDDS.

#### 3.5.5. Effect of Dissolution Media on Drug Dissolution of SNE-L Tablets

**Figure 10:**
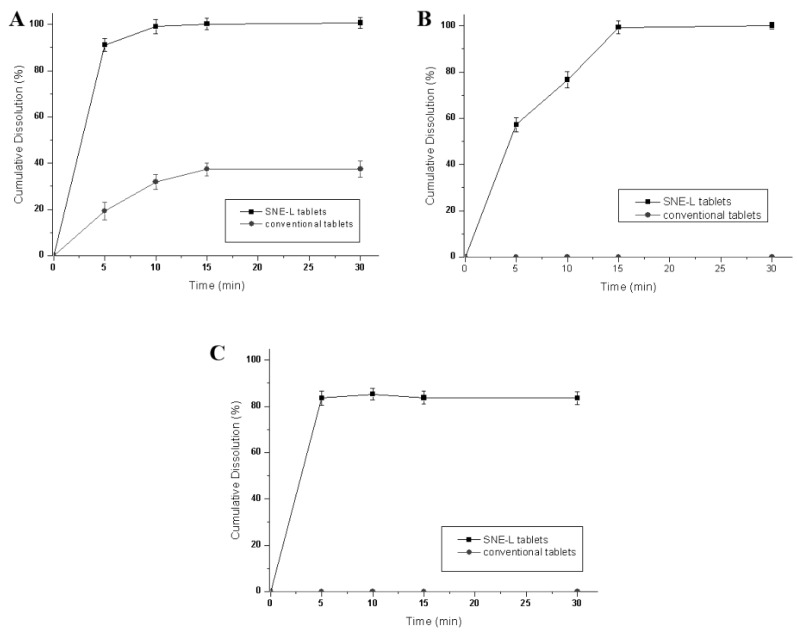
Dissolution profiles of different immediate-release self-nanoemulsifying tablets and conventional tablets in 0.5% SDS solution (**A**); in pH 1.0 hydrochloric acid solution (**B**); and in pH 6.8 phosphate buffer solution (**C**) (*n* = 3).

The dissolution profiles of conventional tablets and solid SNE-L tablets were compared in various aqueous media. In all cases, the dissolution rate of SNE-L tablets was significantly higher than that of conventional immediate release tablets, which can be attributed to the self-emulsifying process as a crucial step preceding drug dissolution. The dissolution profiles in pH 6.8 phosphate buffer solution (Figure 10C) were similar to that in water, and the SNE-L tablets showed a prompt drug release of greater than 80% compared to 0% release for conventional tablets. When the optimal self-emulsifying liquisolid formulation was exposed to 0.5% SDS solution, the degree of dissolution was enhanced up to 100% (Figure 10A) because the added SDS in the media improved the solubility of VK1 [37]. Moreover, pH 1.0 hydrochloric acid solution also facilitated drug release. The acidic media corroded the tablet matrix and dispersed it into smaller particles, which increased the surface area of the drug and enhanced the dissolution percentages of SNE-L tablets. Moreover, the dissolution rate of SNE-L tablets was slower in pH 1.0 hydrochloric acid solution compared with other dissolution media. This slowed dissolution could be due to the presence of acid preventing the release mechanism due to corrosion.

### 3.6. Comparative Pharmacokinetics

**Figure 11:**
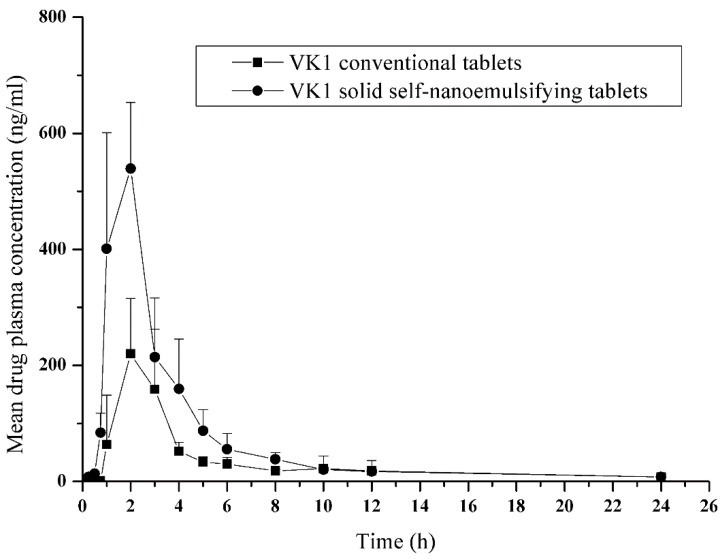
Plasma profile of VK1 in beagle dogs after a single oral administration of solid self-nanoemulsifying VK1 tablets and conventional VK1 tablets (*n* = 6).

The pharmacokinetic profiles of SNE-L tablets and conventional VK1 tablets were compared, and the results are shown in Figure 11. The pharmacokinetic parameters (Table 7) showed that the mean *C*_max_ of SNE-L tablets was 575.46 ± 84.27 ng/mL, which is remarkably higher than that of conventional VK1 tablets (249.23 ± 79.05 ng/mL). Meanwhile, improved rate of VK1 absorption was achieved after oral administration of SNE-L tablets. VK1 could be detected in the plasma of dogs 15 min after the oral administration of SNE-L tablets. In contrast, the plasma concentrations of VK1 could not be detected until approximately 45 min after the oral administration of conventional tablets. Moreover, the AUC of SNE-L tablets was significantly higher than that of conventional tablets. The relative bioavailability of SNE-L tablets compared to conventional tablets was on average approximately 200%. The improved oral bioavailability of VK1 from SNE-L tablets could be attributed to the enhanced dissolution rate in the gastrointestinal tract [30,38,39]. The nanoemulsions formed after self-dispersing in the gastrointestinal tract may enhance drug dissolution through numerous mechanisms, such as increased surface area, improved diffusion, and greater permeability, which ultimately improve the oral bioavailability of SNE-L tablets [28,29].

## 4. Conclusions

In the present study, the SNE-L tablets of VK1 were successfully prepared using SNEDDS and liquisolid technology. VK1 could be released quickly and almost completely from SNE-L tablets. Moreover, the mean *C*_max_ and AUC values of SNE-L tablets were remarkably higher than those of conventional tablets, indicating improved oral bioavailability. In conclusion, the combined application of SNEDDS and liquisolid technology appears to be a promising approach for improving the dissolution and oral bioavailability of oily drug vitamin K1.

## Figures and Tables

**Table 1:** Level codes of factors and corresponding values in central composite design.

Factors	Levels and Corresponding Values				
	−1.414	−1	0	1	1.414
Weight Percent of Oil (X_1_, %)	15	17.2	22.5	27.8	30
K_m_ (X_2_)	0.6	0.95	1.8	2.65	3

**Table 2:** Experimental runs and results of central composite design.

Run	X1	X2	Y1	Y2	Y3
	the Weight Percent of Oil	K_m_	Droplet Size (nm)	Zeta Potential (mV)	DE_20min_
1	22.50	1.80	47.74	−20.53	87.42
2	17.20	0.95	168.87	−23.77	48.72
3	22.50	0.60	230.36	−28.05	31.5
4	17.20	2.65	131.52	−25.21	62.73
5	27.80	2.65	56.35	−24.15	60.79
6	22.50	1.80	50.16	−21.27	93.13
7	22.50	1.80	42.46	−20.65	91.53
8	15.00	1.80	140.19	−24.12	68.12
9	22.50	1.80	44.48	−20.53	93.97
10	22.50	3.00	111.16	−24.47	67.57
11	27.80	0.95	163.39	−28.8	30.02
12	30.00	1.80	69.64	−25.72	50.06
13	22.50	1.80	53.5	−21.06	86.12

**Table 3:** Regression model fitting and ANOVA results for each response.

Model	R-Squared	*p*-Value	Lack of Fit
Y1 = 817.8132 − 39.9592A − 256.9652B − 3.8717AB + 0.9484A^2^ + 82.7705B^2^	0.9929	<0.01	0.1018
Y2 = −54.6640 + 2.4906A + 7.1018B + 0.3383AB − 0.07214A^2^ − 3.7487B^2^	0.9865	<0.01	0.2100
Y3 = −267.9637 + 23.8097A + 100.0931B + 0.9311AB − 0.5905A^2^ − 29.7033B^2^	0.9861	<0.01	0.4842

Y1: Droplet size Y2: Zeta potential Y3: % DE after 20 min.

**Table 4:** Predicted and observed values of a liquid formulation (X1 = 20.0, X2 = 2.0) of L-SNEDDS.

Index of Evaluation	Predicted Values	Observed Values	Error %
droplet size (nm)	60.26	62.58	3.84
zeta potential (mV)	−20.95	−20.31	−3.05
DE_20min_ (%)	90.65	87.96	−2.96

**Table 5:** Φ-value and micromeritic properties of liquisolid powder (*n* = 3).

Liquisolid Powders	Φ-Value	Angle of Repose (°)	Carr’s Index	Hausner’s Ratio
Vivapur^®^ PH102	0.150	44.5 ± 2	34.88 ± 0.21	1.53 ± 0.07
Cellatose^®^ 80	0.125	44.0 ± 2	33.47 ± 0.18	1.47 ± 0.04
Fujicalin^®^	0.714	41.0 ± 3	29.57 ± 0.15	1.32 ± 0.04
Cab-O-Sil^®^ M5P	1.450	43.5 ± 4	35.57 ± 0.25	1.62 ± 0.06
Aeroperl^®^ 300	1.850	40.0 ± 1	28.42 ± 0.16	1.37 ± 0.03

**Table 6:** Liquefaction time of solid self-nanoemulsifying tablets.

Aqueous Media	Liquefaction Time (min)
purified water	8.5 ± 0.8
0.1 N HCl	12.8 ± 0.9
phosphate buffer pH 6.8	8.4 ± 0.7
0.5% SDS aqueous solution	7.9 ± 0.8

**Table 7:** Pharmacokinetic parameters of VK1 in beagle dogs after a single oral administration of solid self-nanoemulsifying VK1 tablets and conventional VK1 tablets (*n* = 6).

Parameter	Solid Self-Nanoemulsifying Tablets	Conventional Tablets
*C*_max_ (ng/mL)	575.46 ± 84.27	249.23 ± 79.05
*t*_max_ (h)	1.67 ± 0.58	2.00 ± 1.00
*t*_1/2_ (h)	5.86 ± 1.19	5.51 ± 2.12
AUC_0−t_ (ng·h/mL)	1682.04 ± 250.56	841.88 ± 210.33
AUC_0−__∞_(ng·h/mL)	1716.33 ± 264.20	866.14 ± 215.45

AUC, area under the curve; *t*_1/2_, halflife.
